# From Videogames to Teaching — Different Camera Perspectives in an Interactive Synchronous Online Tutorial

**DOI:** 10.1007/s40670-023-01833-9

**Published:** 2023-07-20

**Authors:** David Niklas Boten, Nils Daum, Thomas Schutz, Sebastian Spethmann

**Affiliations:** 1 Brandenburg Medical School Theodor Fontane (MHB), Campus Neuruppin, Fehrbelliner Straße 38, 16816 Neuruppin, Germany; 2 Faculty of Health Sciences Brandenburg, Joint Faculty of the University of Potsdam, the Brandenburg University of Technology Cottbus-Senftenberg and the Brandenburg Medical School Theodor Fontane (MHB), Karl-Liebknecht-Str. 24-25, 14476 Potsdam, Germany; 3 Deutsches Herzzentrum der Charité – Medical Heart Center of Charité and German Heart Institute Berlin, Clinic for Cardiology, Angiology and Intensive Care, Campus Charité Mitte, Berlin, Germany; 4 Charité – Universitätsmedizin Berlin, corporate member of Freie Universität Berlin and Humboldt- Universität zu Berlin, Charitéplatz 1, 10117 Berlin, Germany

**Keywords:** Distance education, E-Learning, Educational technology, Clinical skills, Video-based education, First-person video

## Abstract

The global COVID-19 pandemic has required clinical skills training to be transferred to an online format. An interactive synchronous online tutorial with different camera perspectives was developed. In a survey, 79% of the students preferred the first-person perspective, which allowed students to view the abdominal examination through the examiner’s eyes.

## Innovation

The Brandenburg Medical School Theodor Fontane (MHB) is a state-approved private medical university with four campuses in four different cities in the state of Brandenburg. In the MHB skills lab certified by the “Practical Skills Committee” of the “German Association for Medical Education (GMA),” medical students can train their medical skills, e.g., in a peer-assisted learning program. At the end of each semester, these skills are tested in objective structured clinical examinations (OSCEs).

Due to the COVID-19 pandemic, a video-based online tutorial for learning medical skills under corona lockdown conditions was developed as a first test trial. In a systematic review, Youssef et al. show that video-based surgical education is effective in teaching surgical skills [1].

**Fig. 1:**
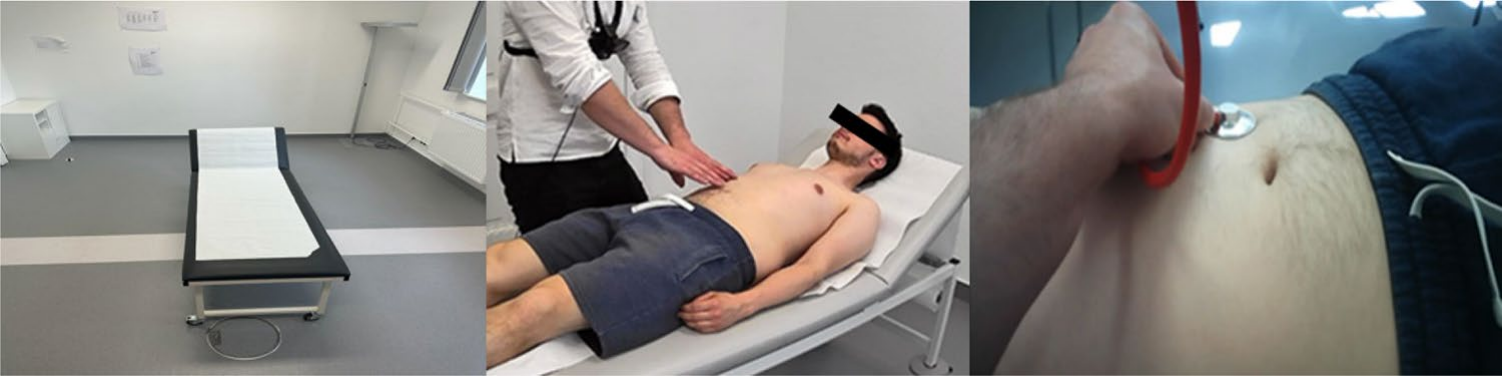
Left: overview perspective of the whole room; center: representation of the oblique overview shot; right: the first-person perspective

An abdominal examination video (and tutorial) was produced by two 6th and 8th grade students (Fig. 1). Both students were student assistants in the skills lab, and both had extensive experience in peer-to-peer teaching. Abdominal examination was selected due to the corona restrictions. Because the abdominal examination is part of the second year curriculum, 19 out of 96 (19.8%) first year medical students were recruited (twelve out of 48 (25%) 1st grade students; seven out of 48 (14.6%) 2nd grade students). Pre-training in examination technique would have resulted in bias.

Three different camera perspectives are offered. The first perspective showed an overall view of the examination room (Fig. 1, left). The second perspective focused on the patient observation during the examination and the examiner’s hands (Fig. 1, center). The third perspective was the first-person perspective (Fig. 1, right). A standard smartphone was used as camera, which was attached to the examiner’s chest with a chest strap (Maclean MC-773 Camera Chest Strap Holder).

The participating students dialed into the virtual classroom (Webex, Cisco Systems, Inc). First, the recorded video of the standardized abdominal examination was shown to avoid bias due to different content. All steps of the abdominal examination were then repeated “live” (via Webex) by the two skills lab tutors. Meanwhile, the students had the opportunity to ask questions. Afterwards, all students had 20 min to practice the examination steps independently from home in teams of two. For this purpose, individual session rooms were set up via Webex. After the self-training period, the shared virtual classroom was opened again, and any questions that arose were discussed in plenary. Afterwards, a preliminary survey was carried out on the preferred camera perspective, before the online tutorial ended after about 90 min. At the end of the semester, the students took their OSCE exam.

First results were presented at the GMA Annual Meeting 2021 [2]. For example, the best OSCE results were obtained in the group of students who participated in the interactive synchronous online tutorial. But most of the workshop discussion related to the different camera positions, especially the first-person perspective. That’s why we decided to share this “innovation” first. The majority (n=15, 79%) preferred the first-person perspective. Some participants (n=3, 16%) preferred a combination of first-person perspective and oblique overview shot. Only one participant (5%) preferred the oblique overview view. The total overview shot was not preferred by anyone. Our results indicate that the first-person perspective is preferred.

This is in line with Burnham et al. [3]: “To best mimic the [face-to-face] student experience, camera position presented the first-person perspective of the faculty member with the faculty member’s hands in view” [3, p. 8]. The first-person perspective is very common in video games, as gamers often prefer this perspective to immerse deeper into the game. Since the majority of 1st and 2nd grade MHB students belonged to Generation Z, the proportion of gamers among the participants was likely to be high.

The innovations’ benefit could lie in incorporating the learner’s digital (gaming) preferences even more into account in their own teaching. Future versions will contain open questions that allow conclusions why most students chose the first-person perspective and what further potentials for improvement the students see. In addition to simple technical on-demand “innovations,” smarter technologies such as 3D and 360° cameras or motion control camera systems will also be used. However, emerging (gaming) technologies might sometimes be able to boost post-COVID healthcare education.

## Data Availability

The datasets generated during and/or analyzed during the current study are available from the corresponding author on reasonable request.

## References

[1] Youssef SC, Aydin A, Canning A, Khan N, Ahmed K, Dasgupta P. Learning surgical skills through video-based education: a systematic review [published online ahead of print]. Surg Innov. 2022:1–19. 10.1177/15533506221120146. 10.1177/15533506221120146 PMC10280671 35968860

[2] Daum N, Boten D, Sendeski M, Schutz T, Spethmann S. Erwerb von Medienkompetenz zur Durchführung eines synchronen Online-Tutoriums zur Entwicklung fachlich-methodischer Basiskompetenzen in der medizinischen Aus- und Weiterbildung. Jahrestagung der Gesellschaft für Medizinische Ausbildung (GMA). [Implementation of a synchronous online tutorial for the development of technical and methodical basic skills in medical education and training. German Association for Medical Education (GMA) Annual Conference 2021] German Medical Science GMS Publishing House 2021. https://www.egms.de/static/en/meetings/gma2021/21gma142.shtml.

[3] Burnham KD, Major CA, Borman WH (2023). First-person video experiences as a vicarious, virtual alternative to in-person basic science labs. J Chiropr Educ.

